# Advancing GCT Management: A Review of miR-371a-3p and Other miRNAs in Comparison to Traditional Serum Tumor Markers

**DOI:** 10.3390/cancers16071379

**Published:** 2024-03-31

**Authors:** Crystal L. Seales, Dhruv Puri, Nuphat Yodkhunnatham, Kshitij Pandit, Kit Yuen, Sarah Murray, Jane Smitham, John T. Lafin, Aditya Bagrodia

**Affiliations:** 1 Morehouse School of Medicine, Atlanta, GA 30310, USA; cseales@msm.edu; 2 Department of Urology, University of California San Diego, La Jolla, CA 92093, USA; dpuri@health.ucsd.edu (D.P.); nyodkhunnatham@health.ucsd.edu (N.Y.); kpandit@health.ucsd.edu (K.P.); k3yuen@health.ucsd.edu (K.Y.); jasmitham@health.ucsd.edu (J.S.); 3 Department of Pathology, University of California San Diego, La Jolla, CA 92093, USA; shmurray@health.ucsd.edu; 4 Department of Urology, University Texas Southwestern Medical Center, Dallas, TX 75390, USA; john.lafin@utsouthwestern.edu

**Keywords:** germ cell tumors, biomarkers, serum tumor markers, miRNAs

## Abstract

**Simple Summary:**

The diagnosis and management of testicular germ cell tumors (TGCTs) currently relies on the use of unreliable biomarkers—conventional serum tumor markers (STMs). Variable levels of elevation in serum samples among patients and cancer types make the use of conventional STMs unfavorable for dependable diagnosis and clinical management of TGCTs. This review of recent studies suggests germ cell tumor associated miRNAs, such as miR-371a-3p, can be utilized as not only a reliable but cost-effective and convenient blood-based biomarker. However, before widespread clinical implementation, the development of a protocol for sample collection, analysis, and interpretation is necessary to prevent overtreatment and guide the management of patients.

**Abstract:**

MicroRNAs, short non-protein coding RNAs, are overexpressed in GCTs. Circulating levels of germ cell tumor (GCT)-associated miRNAs, such as miR-371a-3p, can be utilized as efficient and cost-effective alternatives in diagnosing and managing patients presenting with GCTs. This quality of miRNAs has demonstrated favorable performance characteristics as a reliable blood-based biomarker with high diagnostic accuracy compared to current serum tumor markers (STMs), including α-fetoprotein (AFP), beta human chorionic gonadotropin (β-hCG), and lactate dehydrogenase (LDH). The conventional STMs exhibit limited specificity and sensitivity. Potential clinical implications of miRNAs include impact on de-escalating or intensifying treatment, detecting recurrence at earlier stages, and lessening the necessity of cross-sectional imaging or invasive tissue biopsy for non-teratomatous GCTs. Here, we also highlight the outstanding issues that must be addressed prior to clinical implementation. Standards for measuring circulating miRNAs and determining ideal cutoff values are essential for integration into current clinical guidelines.

## 1. Introduction

The global trend notes a rise in testicular cancer incidence among many different demographics across the globe in recent years [1]. There is a noticeable increase in the occurrence of testicular germ cell tumors (TGCTs), not just in younger cohorts, but notably among individuals over the age of 40 years [2,3]. This malignancy, once categorized as the predominant cancer in younger males, is now impacting the health and well-being of a broader demographic segment than previously recognized [4]. Consequently, the strategies employed by researchers, clinicians, and patients in confronting testicular cancer need to be progressively refined to rise to the challenges posed by the evolving landscape of testicular cancer treatment and management.

Germ cell tumors (GCTs), which arise from abnormal development of germ cells, present in three primary categories: (i) yolk sac tumors and teratomas typically observed in neonates and infants; (ii) seminomas and nonseminomas, more common in adolescents and adults; and (iii) spermatocytic tumors, which are rare but noteworthy [5]. Among these, seminomas and nonseminomas, also referred to as NSGCTs, are of critical concern given that they represent an overwhelming majority—nearly 99%—of testicular germ cell tumor incidences in the 15–44-year age group [1,6]. With the estimated number of new testicular cancer cases in the United States for 2023 being 9120, and with over 291,000 men currently managing this disease, there is a significant impetus to pursue more affordable and less invasive diagnostic and monitoring strategies [7].

The diagnosis of testicular germ cell tumors (TGCTs) predominantly relies on a comprehensive approach that includes a variety of methods to ensure accuracy. These methods typically encompass (i) a thorough physical examination of the testes to detect any abnormalities; (ii) advanced imaging techniques such as ultrasound (US) and magnetic resonance imaging (MRI) to visualize the internal structure of the testes and identify potential tumors; (iii) the measurement of serum tumor marker levels, which serve as a non-invasive liquid biopsy providing preliminary assessment before proceeding to more invasive diagnostic procedures; and (iv) a tissue biopsy post-orchiectomy, the definitive method for diagnosing TGCTs by allowing direct examination of the tumor cells under a microscope [7]. Serum tumor markers are particularly valuable in the diagnostic process, as they involve the analysis of various substances, including metabolites, proteins, and nucleic acids, that are released into the bloodstream by tumor cells, providing insights into changes in gene expression and the genomic profile of the tumor [8]. The traditional serum tumor markers (STMs) used in the diagnosis of TGCTs are α-fetoprotein (AFP), Beta human chorionic gonadotropin (β-hCG), and lactate dehydrogenase (LDH) [9]. However, these conventional STMs have several limitations that can impact their effectiveness in accurately diagnosing TGCTs. One significant limitation is that AFP and β-hCG levels can be elevated in conditions unrelated to TGCTs, such as disruptions in endocrine and metabolic processes. Furthermore, LDH is a marker of cell death and can be elevated in a variety of conditions characterized by increased cell turnover, making it less specific for TGCTs [10,11]. Additionally, seminomas, a type of TGCT, do not produce AFP, so an elevated AFP level would not be indicative of a seminoma but could suggest the presence of non-seminomatous germ cell tumors (NSGCTs). Moreover, both LDH and β-hCG can be elevated in any type of GCT, providing little specificity in determining the exact type of cancer [12]. Studies have highlighted the limitations of conventional STMs in the management of testicular cancer. For example, a study involving 793 patients between January 2014 and July 2021 found that of the 71 patients who had a relapse, only 31 (43.6%) were marker-positive. Additionally, 124 patients (15.6%) experienced at least one false-positive marker elevation, defined as a positive marker result in a patient who remained free from a proven relapse for at least 6 months after the incident. These false-positive results can lead to unnecessary additional imaging or a hesitancy to investigate further due to the perceived irrelevance of the marker elevations [13]. Another study conducted in 2020 reviewed the records of 794 patients with GCTs treated in three Spanish hospitals and found that of the 125 patients who developed a first recurrence, 123 had marker levels recorded. Overall, 79 patients (64%) had elevated tumor markers at diagnosis, and 76 (62%) had elevated markers at first recurrence [14]. The unreliable elevation and lack of specificity of conventional STMs make them less favorable for reliable diagnosis and clinical management. In light of these limitations, there is a growing interest in exploring alternative biomarkers that can provide more accurate and specific diagnostic information. One such promising avenue is the analysis of blood-circulating micro-ribonucleic acids (miRNAs) in standard germ cell tumor screening regimens. These small non-coding RNA molecules have been shown to play a crucial role in gene regulation and have the potential to serve as more reliable and specific biomarkers for the diagnosis and management of TGCTs.

MicroRNAs (miRNAs) are short, non-coding RNA molecules that play a crucial role in the regulation of gene expression, affecting approximately one-third of protein-coding genes in the human genome [15]. The biogenesis of miRNAs involves several steps, starting with the transcription of primary miRNAs (pri-miRNAs) that are subsequently processed into precursor miRNAs (pre-miRNAs). These pre-miRNAs are then further cleaved to form mature miRNAs, which are incorporated into the RNA-induced silencing complex (RISC). Within this complex, miRNAs can bind to complementary sequences in target messenger RNAs (mRNAs), leading to the degradation or translational repression of these target mRNAs [16]. In the context of cancer, miRNA expression can become dysregulated, leading to the disruption of normal cellular processes. miRNAs can act as oncogenes (referred to as oncomiRs) or tumor suppressors, depending on their target genes and the context of their expression. In germ cell tumors (GCTs), oncomiRs are often overexpressed, leading to the suppression of their target tumor suppressor genes. Conversely, tumor suppressor miRNAs can be downregulated, resulting in the overexpression of their target oncogenes [15]. This dysregulation of miRNA levels creates an environment conducive to cancer growth and progression. The alterations in miRNA expression patterns can serve as valuable biomarkers for the detection and differentiation of various histotypes of testicular germ cell tumors [16]. By analyzing these changes, clinicians can gain insights into the molecular underpinnings of the disease, which can inform diagnostic, therapeutic, and prognostic strategies. The application of miRNA-based biomarkers in clinical practice has the potential to enhance the accuracy of diagnosis, guide treatment decisions, and provide prognostic information, ultimately improving patient outcomes in testicular cancer management.

This review aims to illuminate the multifaceted role of circulating microRNAs (miRNAs) as liquid biopsies in the management of germ cell tumors (GCTs). First, we explore the current clinical benefits and implications of utilizing circulating miRNAs, emphasizing their potential to revolutionize non-invasive diagnostic and prognostic approaches in oncology. Second, we delve into the outstanding issues surrounding the use of miRNAs, such as challenges in standardization, quantification, and interpretation, that must be meticulously addressed prior to their widespread clinical implementation. Finally, we discuss the potential applications of circulating miRNAs in GCT management, including their role in early detection, monitoring treatment response, and predicting disease recurrence, thereby underscoring their promise in enhancing patient outcomes and personalizing cancer care.

## 2. Insights into the Advantages of Circulating miRNAs

As tumors proliferate, the constituent cells emit a variety of substances that play a pivotal role in cellular communication [8]. One innovative diagnostic approach, liquid biopsy, involves the collection of bodily fluids—namely blood, urine, or semen—to capture these tumor-derived biomarkers. These specimens are then analyzed using methods tailored to the specific fluid type. The burgeoning interest in liquid biopsies stems from their minimal invasiveness and the consequent ease of sample collection, as well as the method’s potential for continuous monitoring of tumor dynamics [8]. MicroRNAs (miRNAs), in particular, have garnered attention over the past several years. Their stability and profuse presence in the bloodstream render them highly detectable markers, significantly aiding in the identification and tracking of cancerous developments in patients [16].

### 2.1. Discriminating TGCTs from Normal Testicular Tissue

There are numerous clusters of miRNAs associated with specific tumors and their overexpression can assist in accurately predicting cancer histology. For example, MicroRNA-302, which increases the rate of cell proliferation by inhibiting cyclin-dependent kinase (CDK) 2 and 4, is elevated in the blood of TGCT patients, while it is downregulated in liver, stomach, and colon cancer [16]. Of the various clusters of miRNAs considered in making an accurate diagnosis of TGCT, including miR-372-3p, miR-367-3p, miR-371a-3p, and miR-373-3p, it was concluded that quantifying circulating miR-371a-3p alone is sufficient for the diagnosis of TGCT [17]. In addition, variation of miRNA levels between TGCT subtypes has been documented. A study using formalin-fixed paraffin-embedded (FFPE) tissue samples found upregulated miR-371a-3p levels among the TGCT subtypes of pure seminoma, seminoma in mixed TGCT, embryonal carcinoma, and other nonseminomas, including teratomas compared to healthy tissue [18]. It must be noted that pure seminomas exhibited the highest of the miR-371a-3p expression levels, while the pre-pubertal teratomas exhibited the lowest expression level, although still higher than healthy tissue [18]. Currently, there is no reliable circulating miRNA that is available for the detection of teratoma. The levels of miR-371a-3p and the classic GCT markers were also compared in patients with TGCT, and it was found that the sensitivity of the miRNA test was significantly higher than each of the classic markers separately and the markers combined [19]. Dieckmann et al. noted circulating miR-371a-3p levels, also referred to as the M371 test, had a sensitivity of 90.1%, specificity of 94%, and an area under the curve (AUC) of 0.97 when used for the prediction of GCT histology [19,20]. Moreover, three additional investigations corroborated these results, demonstrating sensitivity levels between 93% and 96%, specificity of 100%, and an area under the curve (AUC) ranging from 0.97 to 0.98. Hence, miRNA371 emerges as a potentially valuable novel biomarker for testicular germ cell tumors (TGCT) [21,22,23] (Table 1). Comparatively, serum tumor markers AFP, LDH, and β-hCG had sensitivities of less than 50% in seminomas.

### 2.2. Treatment Monitoring

Common treatment options for testicular germ cell tumors range from orchiectomy alone to radiotherapy, chemotherapy, or retroperitoneal lymph node dissection (RPLND) [24]. While it is possible for TGCT cure rates to be high, the use of the mentioned treatments, either independently or in combination, can cause toxic effects resulting in treatment related morbidity [25]. Complications can include but are not limited to cardiovascular disease, additional malignant neoplasms, pulmonary complications, nephrotoxicity, and avascular necrosis [26]. Using miRNAs to verify treatment effectiveness can assist healthcare providers in determining or revising the patient-centered treatment plan to decrease excessive procedures and exposure to risks. When necessary, miRNAs have the potential to identify germ cell tumors that are resistant to standard treatments. A study by Leão et al. documented a significant decrease in miRNA levels from pre-chemotherapy to post-chemotherapy [27]. Circulating levels of miR-371a-3p also correctly predicted residual GCTs post-chemotherapy with 100% sensitivity. In addition, a decrease in miRNA was observed following orchiectomies; however, levels did not return to a normal range [19]. Access to these data can support or oppose options for retroperitoneal lymph node dissection, which is documented to be therapeutic in about 55–60% of patients after chemotherapy [27]. Surveillance after treatment also compounds potential treatment risks related to 6–10 additional imaging scans and exposure to ionizing radiation [28,29]. Considering the noted sensitivity and specificity of miR-371a-3p circulating levels, the possibility of deescalating the use of unnecessary treatment or surveillance methods can become a reality [29].

### 2.3. Detection of Early Relapse

The difference observed in miR-371a-3p elevation between TGCT subtypes is also illustrated between clinical stages. In a study containing 874 participants between the of ages 16 and 69 years, 616 with TGCTs and 258 controls, concluded that patients of a clinical stage greater than 1 (one) had higher miR-371a-3p levels than those with clinical stage 1 (CSI) [19]. More advanced staged tumors consistently expressed a higher median of miRNA values than that of localized tumors. This is explained by the likelihood of the miRNA levels correlating with the physical amount of tumor present in the body [19]. This described classification of “high burden” relapse correlated with significantly higher miR-371a-3p levels compared to that found in low burden relapse. With the highest risk of relapse occurring during the first 2–3 years after initial treatment, about 30% of patients on active surveillance will relapse during follow-up [30]. The retroperitoneum was found to be one of the most common sites of relapse. Diagnosis of relapse sooner in the disease process increases the chance of cure by treatment. With knowledge of likely sites of relapse and metastasis, detection approaches must meet the demand for precision. CT scans or radiography usually only detect metastases about ≥1 cm in size causing tumor progression to initially go unnoticed. In addition, imaging at institutions can occur at a frequency ranging from every 2 months after initial staging and diagnosis to eventually every year, once again increasing the potential radiation burden [30]. A study by Hamilton et al. illustrated a large discrepancy between the current inconsistent methods of relapse detection. This study observed relapse was detected by routine imaging in about 52% of cases, STMs in 33% of cases, and only 11% of cases were first detected by both positive imaging and STMs simultaneously [30]. During active surveillance, the change in miRNA levels can predict relapse at initial stages. miR-371a-3p values were found to be higher at relapse compared to postorchiectomy levels for 94.1% of patients in the Lobo et al. investigation [31]. Moreover, 100% of patients exhibiting recurrence in the Fankhauser et al. study were determined to have elevated miR-371a-3p levels. Of those patients, 80% of relapse was identified with marked miRNA levels before the use of standard imaging and tumor marker elevation [32]. Monitoring miR-371a-3p levels decreased recurrence detection time by upward of 2 months compared to standard follow-up methods including imaging [32].

## 3. Current Limitations

### 3.1. Expression of miRNAs Differ among TGCT Subtypes

A generalized miRNA serum cut-off value for TGCTs has not been established in part due to the documented variation in levels among TGCT subtypes. The difference in levels provides a greater challenge in interpreting the resulting values and translating the acquired information into a usable clinical decision-making schema. Vilela-Salgueiro et al. described the relative levels of expression in GCT subgroups. Seminomas have the greatest increase in miRNA expression compared to non-seminomatous TGCTs. Comparatively, pre-pubertal TGCTs have lower expression levels than post-pubertal TGCTs [18]. It is also notable that only 59% of seminomas less than 1 cm in size express markers making them more difficult to detect [19]. Teratomas do not express miR-371a-3p or express less than other subtypes [19]. Proper distinction and categorization of lab values that accurately reflect disease burden and subtypes are necessary to appropriately determine treatment plans [18]. Variation in expression levels can lead to more specific therapies, but concurrent limitations such as methodological factors make this information use premature. This matter is also complicated by the lack of elevated miRNA expression, such as miR-371a-3p, in testicular tumors not originating from germ cells [33]. With this consideration, a greater use of serial miRNA measurements with standardized protocols could reveal more information on expression levels among various populations of patients diagnosed with TGCTs. 

### 3.2. Unrefined Standardized Protocols 

Understanding the immense possibilities for use of circulating microRNAs is underway; however, a major hindrance could result from the current lack of standardized conventions used to collect, store, analyze, and interpret data. The development of an accepted protocol may limit obstacles, such as interlaboratory heterogeneity, allowing for widespread clinical use of circulating miRNA levels [34,35]. Current studies are gaining traction on this issue [28]. Lafin et al. suggested the use of raw Cq values from serum qPCR-based methods with pre-amplification after discovering the normalization process of assays increased the likelihood of variation and therefore heterogeneity [34]. The use of raw miR-371a-3p Cq data, in this context, is associated with lower cost, which can be useful for incorporation of miRNA analysis into routine clinical testing. Baseline decision-making criteria aimed at reducing misclassification of samples via establishment of ideal cut-off values for negative, positive, or indeterminate (grounds for reanalysis) samples are also suggested in the mentioned study. Cut-off values may be dependent on the clinical scenario as the expression of miRNA varies between disease subtypes as previously described. Further, each lab may have variable cutoffs as well. Protocol formation can prevent overtreatment and guide the management of patients.

## 4. Potential Applications of Circulating mRNAs

### 4.1. Risk Assessment and Genetic Screening

Individuals predisposed to common cancers such as breast cancer and colon cancer make up an estimated 5–10% of cancer occurrences [36]. This phenomenon, termed hereditary cancer, results from germline mutations in certain predisposition genes, such as the breast cancer susceptibility gene (*BRCA1/2)* in breast cancer and the MutS homolog 2 (*MSH2)* gene in colon cancer [36]. Knowledge and identification of these common cancer syndromes rely heavily on the availability and efficacy of genetic tests. Risk assessment and genetic screening for TGCTs can lead to more informed decision-making for at-risk individuals who may also be at higher risk of developing more than one primary cancer. Evaluation and development of certain miRNA profiles could be used to assess an individual’s probability of developing germ cell tumors, allowing for preventative measures.

TGCTs arise from various factors including environmental and genetic susceptibilities. However, familial aggregation of cancer is greatly due to inherited susceptibility in the absence of strong environmental risk factors [37]. A sibling is at a four-fold increase for colon cancer if another sibling is diagnosed [37]. A two- to three-fold risk is illustrated for breast cancer in daughters who had a mother and sister affected with breast cancer [37]. This increase in risk due to heritable factors is also observed in various iterations for those diagnosed with testicular cancer: (i) a four-fold increase in sons with fathers who have testicular cancer [38], (ii) an eight-fold increase when a brother has testicular cancer [38], and (iii) a three-fold increased risk for first-degree relatives of patients with testicular cancer [39] (Table 2). Although low penetrance for testicular cancer is supported, this finding suggests the polygenic nature of this disease process [37]. Each genetic hit or mutation acts in synergy to increase the odds of developing cancer. 

With the availability and development of different miRNA profiling methods, screening could highlight the presence of malignant cells. These alterations, which can be explained by a variety of mechanisms including epigenetic changes, DNA point mutations, and chromosomal modifications of genes, are illustrated by the differences in miRNA expression [40]. The relationship between miRNA dysregulation and cancer is unlikely to be random. This was supported by data suggesting that at least 50% of miRNAs are located at disturbed genomic sites involved in cancer [41]. This has been demonstrated with the 13q31 region found to be amplified in B-cell lymphomas, which includes the miR-17-92 cluster of seven miRNAs, resulting in a 10-fold increase in pri-miRNA levels in 65% of B-cell lymphoma samples [42]. In parallel, the TGCT-related miRNAs exhibiting increased expression, such as miR-371a-3p, are found in the 19q13.42 region [43]. In addition to TGCTs-related miRNAs, significant recurring mutations were observed in the *KIT*, *KRAS*, and *NRAS* genes of the seminoma subtypes at rates of 18%, 14%, and 4%, respectively [44]. The mentioned genes, if mutated, can result in constitutive action of their protein products, leading to heightened stimulation of signaling pathways and therefore cell proliferation [45]. The variation of miRNA gene expression in combination with recently found gene associations such as the *KRAS* gene found on chromosome 12 (i12p) in TGCTs can lead to a comprehensive genetic profile, suggesting a risk of cancer and potential tumor types and subtypes.

Once a patient is evaluated for their known or suspected risk of GCTs, the subsequent actions involving genetic counseling are vital for supporting the patient. Just as innovations in technology allow for advanced screening at costs more affordable for the general public, the same is occurring within the evolving field of cancer genetic counseling. What once was ladened with exorbitant costs and strict insurance-based criteria is now more accessible to a broader set of patients as it plays a significant role in the management of care [46]. Patients and providers can easily obtain a snapshot of the general risk of developing common cancers, including breast, ovarian, and colon cancer, with the use of online databases and risk assessment tools. This knowledge of mutation probabilities depends on standardized testing strategies and an investment in research that provides a foundation for subsequent streamlined clinical biomarkers and genetic screenings [47]. Incorporating miRNA biomarker levels enhances the current risk stratification categories, especially for TGCTs that currently include several environmental and genetic factors such as age, ethnicity, cryptorchidism, and dysregulation of the *KIT* gene [48]. Patients also deemed as exceptional candidates for screening include those mentioned above with a family history of disease. Genetic testing can reduce morbidity and mortality [49]. With this information, more providers are recommending appropriate screening options for eligible participants. Most patients screened are satisfied in choosing to do so. A BRCAsearch study from 2019 reported that 98.7% of participants receiving *BRCA1/2* germline mutation testing were content with having pursued the testing [50].

### 4.2. Early Detection Biomarkers

MiRNAs could serve as early biomarkers for the presence of germ cell tumors in patients not previously diagnosed, enabling earlier identification and staging of the disease. With the increase in screening and possible diagnoses, there is an understandable burden of knowledge placed upon patients, screening participants, and their social support. Mellon et al. describes the risk perception and cancer worries among relatives associated with familial breast cancer risks [51]. The level of worry related to inherited cancer risks supports the need for cancer genetic counseling in addition to reliable means for evaluating biomarkers to provide patients with a realistic and complete picture of their health options. 

Clinical stage I (CSI) seminoma and nonseminoma testicular cancer accounts for 68% of cases in newly diagnosed patients. As the most common stage of disease, it is defined as being confined to the testis without evidence of disseminated disease [52,53]. It is important to note that early detection in the majority of patients does not equate to early action, especially when active surveillance is a recommended option absent of treatment related consequences [53]. Discovery of disease at such an early stage allows the patient and their family to have more control over their future treatment decisions. Access to individualized treatment plans appropriately matched to specific diagnoses will contribute to an improved quality of life. On average, from the development of symptoms to definitive diagnosis, there is a delay from 3 to 5 months, but diagnostic delay can range from 1 to 36 months, resulting in clinical consequences [54]. The detection of TGCTs in CSI has a 5-year survival rate of 99%, while later stages have a decreased survival rate of 72.5% [52]. The use of early detection and active surveillance becomes a greater priority in the clinical setting under these circumstances, which has demonstrated reduced quantities of surgeries, chemotherapy cycles, and radiation treatments with similar survival rates [53].

## 5. Conclusions

Investigation of miRNAs in relation to germ cell tumors is gaining traction and is documented to be superior to conventional tumor markers. The use of specific circulating markers such as miR-371a-3p gives insight into patients’ disease status, treatment efficacy, and recurrence in a non-invasive, cost-effective manner. The current benefits and additional applications are, however, met with weaknesses that must be addressed before the full breadth of potential for this liquid biopsy test is realized. Establishing a standardized methodology for providing valid and reliable results could lead to the utilization of differences seen among TGCT subtypes. MiRNA values can improve the clinical decision-making process for physicians in the care of patients diagnosed with germ cell tumors. Enhanced detection, diagnostic mechanisms, and potentially preventive strategies for cancer bring about new ethical and personal challenges [55]. Although widespread early detection mechanisms for GCTs such as miRNA biomarkers are being explored, the question of equitable access to these screening methods and the influence of outside sources (socio-economic, cultural, and political) act as potential barriers in its implementation and warrant future inquiry. 

## Figures and Tables

**Table 1:** miR371 for TGCT primary diagnosis.

Author	Year	Patient (n)	Sensitivity (%)	Specificity (%)	AUC *
Dieckmann [19,20]	2019	616	90.1	94	0.97
Nappi [21]	2019	110	96	100	0.97
Badia [22]	2021	69	93	100	0.98
Sequeira [23]	2022	82	94	100	0.98

* Note: AUC = area under the curve.

**Table 2:** Familial risk factors associated with testicular germ cell tumors.

Diagnosed Relative	SIR *	Reference
Father	4	[38]
Brother	8.46	[38]
Son	4.5	[38]
First-degree relative	3.1	[39]

* Note: SIR = standardized incidence ratios.
